# Syn5 RNA polymerase synthesizes precise run-off RNA products

**DOI:** 10.1093/nar/gkt1193

**Published:** 2013-11-26

**Authors:** Bin Zhu, Stanley Tabor, Charles C. Richardson

**Affiliations:** Department of Biological Chemistry and Molecular Pharmacology, Harvard Medical School, 240 Longwood Avenue, Boston, Massachusetts 02115, USA

## Abstract

The enzyme predominantly used for *in vitro* run-off RNA synthesis is bacteriophage T7 RNA polymerase. T7 RNA polymerase synthesizes, in addition to run-off products of precise length, transcripts with an additional non-base-paired nucleotide at the 3′-terminus (N + 1 product). This contaminating product is extremely difficult to remove. We recently characterized the single-subunit RNA polymerase from marine cyanophage Syn5 and identified its promoter sequence. This marine enzyme catalyses RNA synthesis over a wider range of temperature and salinity than does T7 RNA polymerase. Its processivity is >30 000 nt without significant intermediate products. The requirement for the initiating nucleotide at the promoter is less stringent for Syn5 RNA polymerase as compared to T7 RNA polymerase. A major difference is the precise run-off transcripts with homogeneous 3′-termini synthesized by Syn5 RNA polymerase. Therefore, the enzyme is advantageous for the production of RNAs that require precise 3′-termini, such as tRNAs and RNA fragments that are used for subsequent assembly.

## INTRODUCTION

RNA plays fundamental roles in cell physiology and is an important target for biomedical research and biotechnology. RNA transcripts synthesized by RNA polymerase *in vitro* are used widely in applications that include hybridization analysis, NMR and crystallographic structural studies, biochemical and genetic studies, and the preparation of functional molecules such as tRNA, mRNA, sRNA, ribozymes and aptamers.

The RNA polymerase encoded by bacteriophage T7 is used widely to synthesize RNA molecules (1–5). These reactions use DNA that contains a T7 RNA polymerase promoter to initiate synthesis. RNA synthesis proceeds to the end of the DNA, resulting in a ‘run-off synthesis’ product. The other two enzymes available for run-off RNA synthesis are bacteriophage T3 and SP6 RNA polymerase (1,6–8), which have properties similar to those of T7 RNA polymerase. Problems encountered with these RNA polymerases include limited processivity, high salt sensitivity (9), undesired products resulting from abortive synthesis (10) and most significantly, the addition of a non-base-paired nucleotide at the 3′ end of the run-off transcript (4,6). This latter product is designated N + 1 product (11). The N + 1 product is usually 50–200% of the desired RNA transcript depending on the reaction conditions (12,13). Extensive efforts have been made to improve the 3′ homogeneity of T7 transcripts including modification of the DNA templates (12,13) and the attachment of ribozymes to the 3′ end of the desired RNAs (14–16). These methods are partially effective but increase the cost and the complexity of the process. Consequently an RNA polymerase reaction that would yield precise, homogeneous run-off products would offer a significant advantage over existing methods.

Bacteriophages are the most abundant and diverse biological entities on earth. Recently, genome sequencing and bioinformatics studies revealed marine phages to be the numerically largest and most diverse group of known organisms in the ocean. Phages that infect the dominant cyanobacteria from the genera *Synechococcus* and *Prochlorococcus* are estimated at 10^30^ particles in the oceans (17). About 60–80% of their putative proteins have no sequence similarity to known proteins in the database. Since a large portion of these proteins must play roles in nucleic acids metabolism, one would expect numerous novel mechanisms underlying the fundamental processes including transcription, DNA replication and recombination. Phage enzymes have played critical roles in biochemical research and biotechnology as reagents for DNA/RNA processing. Biotechnology requires diverse and efficient molecular tools for nucleic acid manipulation and phage proteins are always good candidates due to their simplicity and high efficiency. However, biochemical characterization of phage proteins has been largely limited to phages identified during the onset of molecular biology when only a tiny portion of the huge phage group had been revealed. Consequently the popular phage protein tools are mostly derived from very limited types of phages found in similar environments. We anticipate that studies on novel marine phages will reveal enzymes with properties amenable for use as research tools.

We have recently characterized the first single-subunit RNA polymerase isolated from marine organisms (18). The cyanophage Syn5 (19) RNA polymerase recognizes a unique 15 bp promoter sequence. Using homogeneous recombinant protein, we have established an *in vitro* Syn5 transcription system and investigated the properties of the enzyme and its products. Syn5 RNA polymerase has several advantages over T7 RNA polymerase in synthesizing RNA from linear DNA templates. These advantages include the recognition of a relatively short promoter sequence, a high tolerance to salt and high processivity. However, the most significant advantage of the Syn5 enzyme is the much higher homogeneity of the 3′-termini of its RNA products. RNA synthesis catalysed by Syn5 RNA polymerase results in precise run-off with the products lacking non-based additional nucleotides. The N + 1 product synthesized by T7 RNA polymerase cannot be removed by routine gel extraction and this impedes the function of these RNAs in applications where the precise 3′-terminus of the RNA is critical. These applications include the synthesis of tRNA molecules, RNA probes, RNA primers, genomes of some RNA viruses, RNAs for ligation and assembly, and specific RNAs for structure studies. Thus we believe that Syn5 RNA polymerase will be a valuable tool to generate RNAs with improved functionality in these applications.

## MATERIALS AND METHODS

### Materials

Oligonucleotides were obtained from Integrated DNA Technology. DNA purification kits and Ni-NTA resin were from Qiagen. Preparative Superdex S200 for gel filtration was from GE Healthcare. Restriction endonucleases, T4 DNA ligase, DNase I, RNase I and T7 RNA polymerase (50 U/µl, 2 µM) were from New England Biolabs. Radiolabeled nucleotides were from Perkin Elmer. RNA Clean & Concentrator™-5 kit was from ZYMO Research. RNaseOUT™ Recombinant Ribonuclease Inhibitor was from Invitrogen. Yeast inorganic pyrophosphatase was from Sigma-Aldrich. Nucleoside-5′-triphosphates were from USB except for 5-methylcytidine-5′-triphosphate and pseudouridine-5′-triphosphate that were from Trilink. *Escherichia coli* total aminoacyl-tRNA synthetases were from Sigma-Aldrich.

### Protein purification

His-tagged Syn5 RNA polymerase was produced from a pET24 vector harboring the His-tagged Syn5 RNA polymerase gene between the NdeI and NotI sites (18) and purified by Ni-NTA agarose and gel filtration chromatography. NaCl (2 M) was present during the purification procedure and was removed by dialysis during the final concentration of the purified protein. *Escherichia coli* BL21(DE3) cells over-producing the protein were cultured in 2 L LB medium containing 50 µg/ml kanamycin at 37°C until they reached an OD_600_ of 1.2. The gene for Syn5 RNA polymerase was induced by the addition of 0.5 mM IPTG at 16°C and incubation was continued for 3 h. The cells were harvested, resuspended in 50 mM sodium phosphate, pH 8.0, 100 mM NaCl and lysed by three cycles of freeze-thaw in the presence of 0.5 mg/ml lysozyme. NaCl at a final concentration of 2 M was then added to the lysed cells and the cleared lysate was collected by centrifugation. Ni-NTA agarose (2 ml) was added to the clear lysate and gently mixed at 4°C overnight. The resin was then washed with 30 ml of Wash Buffer (50 mM sodium phosphate, pH 8.0, 2 M NaCl and 10 mM imidazole). Syn5 RNA polymerase was eluted from the column using a 60 ml gradient (20–250 mM imidazole) in Wash Buffer. The majority of His-tagged Syn5 RNA Polymerase eluted at 20–100 mM imidazole. Fractions were analysed on SDS-PAGE gels and those in which Syn5 RNA polymerase was at least ∼80% of the total protein (40–100 mM imidazole elution) were collected and concentrated with Amicon Ultra-15 Centrifugal Filter Units (Millipore) to 1 ml. The concentrated sample was directly loaded onto a 200 ml preparative Superdex S200 column. The gel filtration buffer contained 20 mM Tris-HCl pH 7.5, 2 M NaCl, 0.5 mM DTT and 0.5 mM EDTA. Fractions were analysed on SDS-PAGE gels and those fractions that contained homogenous Syn5 RNAP were pooled. The pooled fractions were concentrated by Amicon Ultra-15 Centrifugal Filter followed by dialysis against Final Dialysis Buffer (50 mM Tris-HCl pH 8.0, 100 mM NaCl, 20 mM β-ME, 1 mM EDTA, 50% glycerol and 0.1% Triton® X-100) and stored at –20°C. Dilutions for enzyme assays were made using the Final Dialysis Buffer. In this buffer the protein remains soluble at concentrations up to 400 µM and we did not try to concentrate the protein further. The yield of His-tagged Syn5 RNA polymerase following this procedure was 5 mg protein per gram of wet cells. His-tagged T7 RNA polymerase was purified from *E. coli* BL21(DE3) harboring plasmid pET28a with the T7 RNA polymerase gene inserted between the NdeI and HindIII sites, using Ni-NTA and DEAE ion-exchange chromatography.

### DNA templates for transcription assays

**Figure 1.**
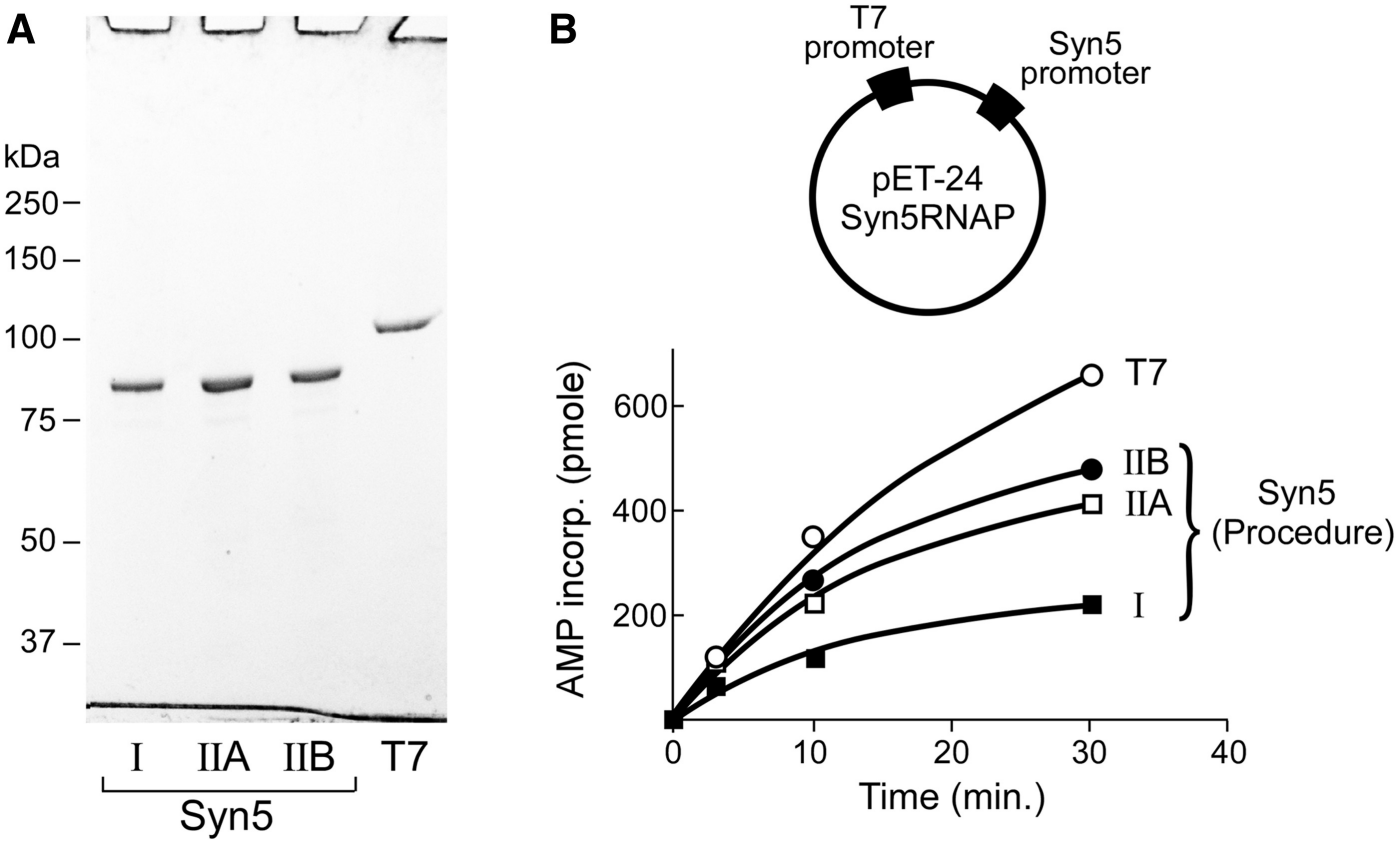
RNA polymerases used in this study. (**A**) SDS-PAGE gel of Syn5 RNA polymerase purified as previously reported (Syn5 I, 18), newly purified Syn5 RNA polymerase using Ni-NTA chromatography in the presence of 2 M NaCl (Syn5 IIA), newly purified Syn5 RNA polymerase purified using Ni-NTA and gel filtration chromatography in the presence of 2 M NaCl (Syn5 IIB) and T7 RNA polymerase. (**B**) Incorporation of AMP at 24°C by 50 nM of each of the RNA polymerases shown in (A). A plasmid with both a Syn5 and T7 promoter was used as the template; each RNA polymerases uses its cognate promoter exclusively to initiate transcription.

**Figure 2.**
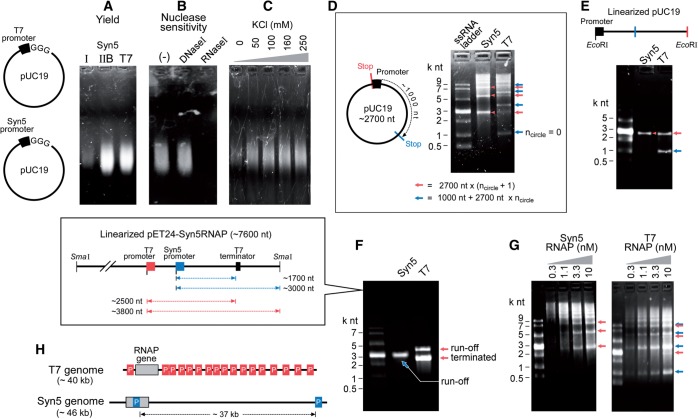
Apparent processivity of Syn5 and T7 RNA polymerases. (**A**) Agarose gel showing RNA synthesis by previously purified Syn5 RNA polymerase (I), newly purified Syn5 RNA polymerase using the improved procedure (IIB) and T7 RNA polymerase on a plasmid template containing a single Syn5 or T7 promoter, as indicated in the schematics at the left. (**B**) The product shown in (A) synthesized by Syn5 RNA polymerase IIB was incubated with 1 U/µl RNase I or DNase I to confirm that it contained RNA. (**C**) Effect of increasing KCl concentration on transcription by Syn5 RNA polymerase. (**D**) Products synthesized by Syn5 and T7 RNAP in a rolling-circle reaction were analysed under denaturing conditions. (**E**) Same assay as in (D) except that the plasmid templates were linearized as shown in the schematic. (**F**) Run-off RNA synthesis by Syn5 and T7 RNA polymerase on template containing a T7 terminator sequence (see schematic). (**G**) The effect of decreasing concentration of T7 and Syn5 RNA polymerase on the processivity of transcription. (**H**) RNA polymerase promoter distribution on coliphage T7 and cyanophage Syn5 genomes.

DNA templates for transcription assays were either plasmids or dsDNA fragments constructed using synthesized oligonucleotides. To test the processivity of Syn5 and T7 RNAP, a single Syn5 or T7 promoter containing three guanosine residues downstream of the promoter (Syn5 5′-**ATTGGGCACCCGTAA**GGG-3′ or T7 5′-**TAATACGACTCACTATA**GGG-3′) was inserted between the BamHI and XbaI sites of plasmid pUC19 to form pUC19-S5P or pUC19-T7P. The templates for run-off tRNA^Arg^ synthesis were formed by annealing complementary synthesized oligonucleotides for each of the two sequences: S5P-tDNAR: 5′-GCC**ATTGGGCACCCGTAA**GCATCCGTAGTTCAGCTGGATAGAGTACTCGGCTACGAACCGAGCGGTCGGAGGTTCGAATCCTCCCGGATGCACCA-3′ and T7P-tDNAR: 5′-GCC**TAATACGACTCACTATA**GCATCCGTAGTTCAGCTGGATAGAGTACTCGGCTACGAACCGAGCGGTCGGAGGTTCGAATCCTCCCGGATGCACCA-3′. These two templates are identical except for their promoter region. The plasmid pET24 harboring the Syn5 RNA polymerase gene (pET24-S5RNAP) containing a single Syn5 promoter in the RNA polymerase gene and a single T7 promoter preceding the gene was used for the filter binding assay shown in Figure 1. pET24-S5RNAP DNA was linearized by digestion with SmaI or NotI and purified by gel extraction to use as a template for the experiment shown in Figures 2F and 5A, respectively. The templates for examination of the nucleotide preference for the initiation of RNA synthesis contain a Syn5 promoter followed by a 37 nt template and its variants as noted in Figure 6. They were constructed by annealing complementary synthesized oligonucleotides. Their non-template strands have sequences: 5′-GGT**ATTGGGCACCCGTAA**G^+1^(or A^+1^ or C^+1^ or T^+1^) G^+2^(or A^+2^ or C^+2^ or T^+2^) AGAACCTTAAGGTTTAACTTTAAGACCCTTAAGTG-3′.

### Transcription assays

To compare the specific activities of purified RNA polymerases (Figure 1B), reaction mixtures containing 40 mM Tris-HCl pH 8.0, 2 mM spermidine, 10 mM DTT, 200 µM GTP, CTP, UTP and ^3^H-ATP (20 cpm/pmole), 1.5 U/µl RNaseOUT^TM^, 50 nM Syn5 or T7 RNA polymerase and 4 nM plasmid pET24-S5RNAP were incubated at 24°C. After various times the reactions were stopped by the addition of 20 mM EDTA. The mixtures (4 µl) were then loaded onto Whatman DE81 filter paper disks and the disks were washed to remove unincorporated ^3^H-ATP. The amount of bound ^3^H-AMP, corresponding to nucleotides incorporated into newly synthesized RNA, was measured using a scintillation counter. The data were analysed using Prism software.

Reaction mixtures (10 µl) for the comparison of apparent processivity of Syn5 and T7 polymerases (Figure 2) contained 40 mM Tris-HCl pH 8.0, 6 mM MgCl_2_, 2 mM spermidine, 10 mM DTT, 600 µM ATP, GTP, CTP and UTP (600 µM is the optimal concentration for rNTPs when 0.04 U/µl yeast inorganic pyrophosphatase is present), 1.5 U/µl RNaseOUT^TM^ recombinant ribonuclease inhibitor, 0.04 U/µl yeast inorganic pyrophosphatase, 10 nM pUC19-Syn5P3G or pUC19-T7P3G, 10 nM Syn5 or T7 RNA polymerase and varying amounts of KCl as indicated in the figure and described in the figure legend. After incubations for 1 h at 24°C, one unit of DNase I was added to each reaction mixture and incubated for an additional 15 min at 37°C to remove the DNA templates. For Figure 2A–C, 2 µl of each reaction mixture was mixed directly with native loading dye and loaded onto a 1.6% TAE agarose gel. For Figure 2D–F, RNA products were purified with RNA Clean & Concentrator™-5 kit into a final volume of 10 µl and 5 µl of each was mixed with 5 µl denaturing RNA loading dye (New England Biolabs). Samples were then heated at 65°C for 5 min and loaded onto a 1.6% TAE agarose gel. RNA products were visualized by staining with ethidium bromide.

**Figure 3.**
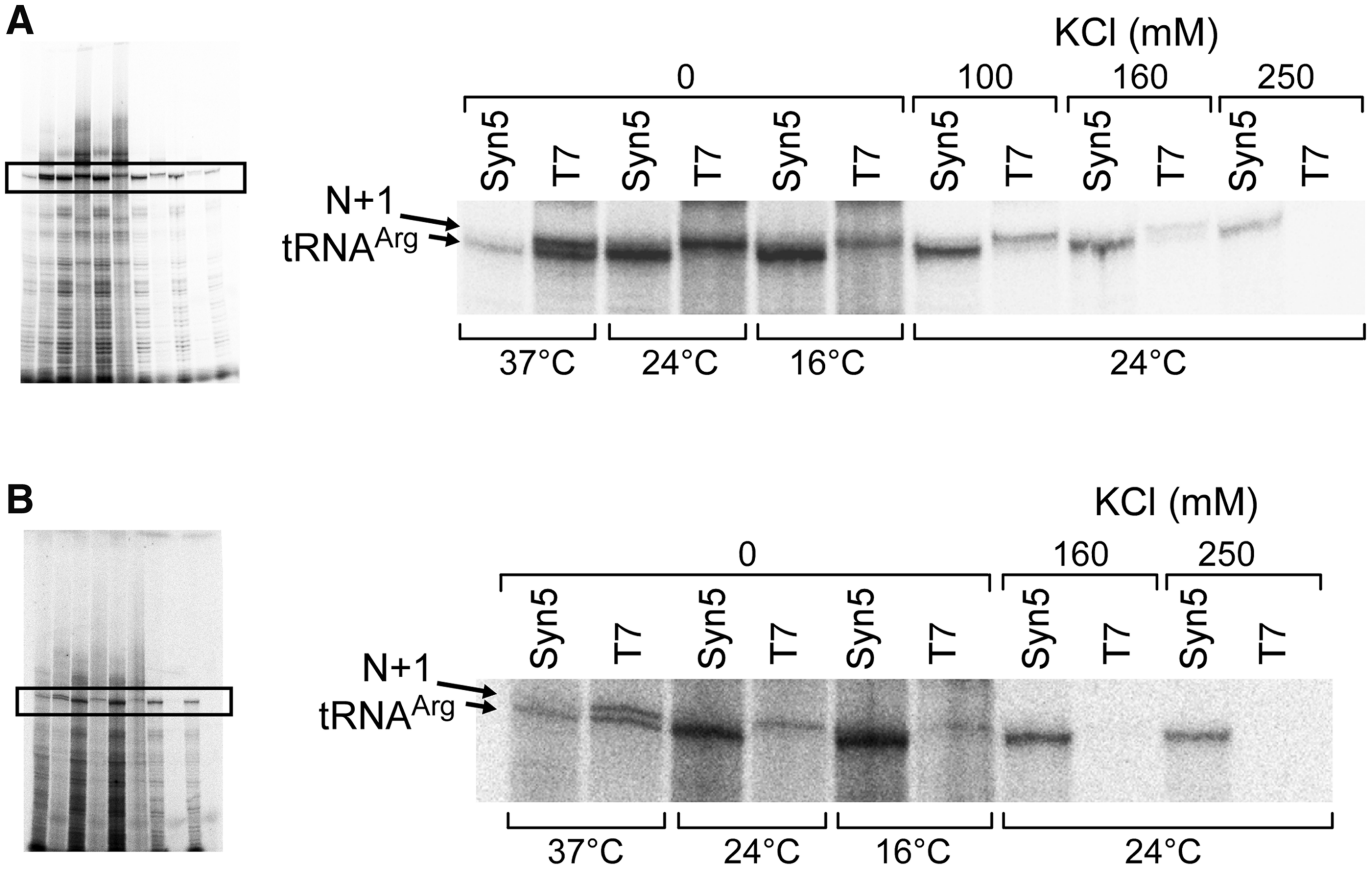
Comparison of T7 and Syn5 RNA polymerase in run-off synthesis of small RNA. (**A**) Effect of temperature and salt concentration on RNA synthesis by T7 and Syn5 RNA polymerase. The region of the predicted product and the N + 1 product is enlarged from the region in the black rectangle from the whole gel. (**B**) The same experiment as described in (A) except that His-tagged T7 RNA polymerase was used.

In order to compare tRNA synthesis by Syn5 and T7 RNA polymerases (Figure 3), reaction mixtures (10 µl) contained 40 mM Tris-HCl pH 8.0, 6 mM MgCl_2_, 2 mM spermidine, 10 mM DTT, 200 µM ATP, GTP and UTP, 10 µM [α-^32^P]CTP, 1.5 U/µl RNaseOUT^TM^ recombinant ribonuclease inhibitor, 0.5 µM DNA templates S5P-tDNAR or T7P-tDNAR, 100 nM Syn5 or T7 RNA polymerase, and varying amounts of KCl as noted in the figure were carried out at either 16, 24 or 37°C for 30 min before adding of one unit of DNase I to each reaction mixture, and then incubated for an additional 15 min at 37°C. Reactions were then terminated by the addition of 4 µl of loading dye containing 95% formamide and 40 mM EDTA. Samples were then heated at 90°C for 1 min and 4 µl of each sample was loaded onto a 15% TBE-Urea denaturing polyacrylamide gel. After electrophoresis, gels were dried and the radioactivity was analysed using a Fuji BAS 1000 Bioimaging Analyzer.

**Figure 4.**
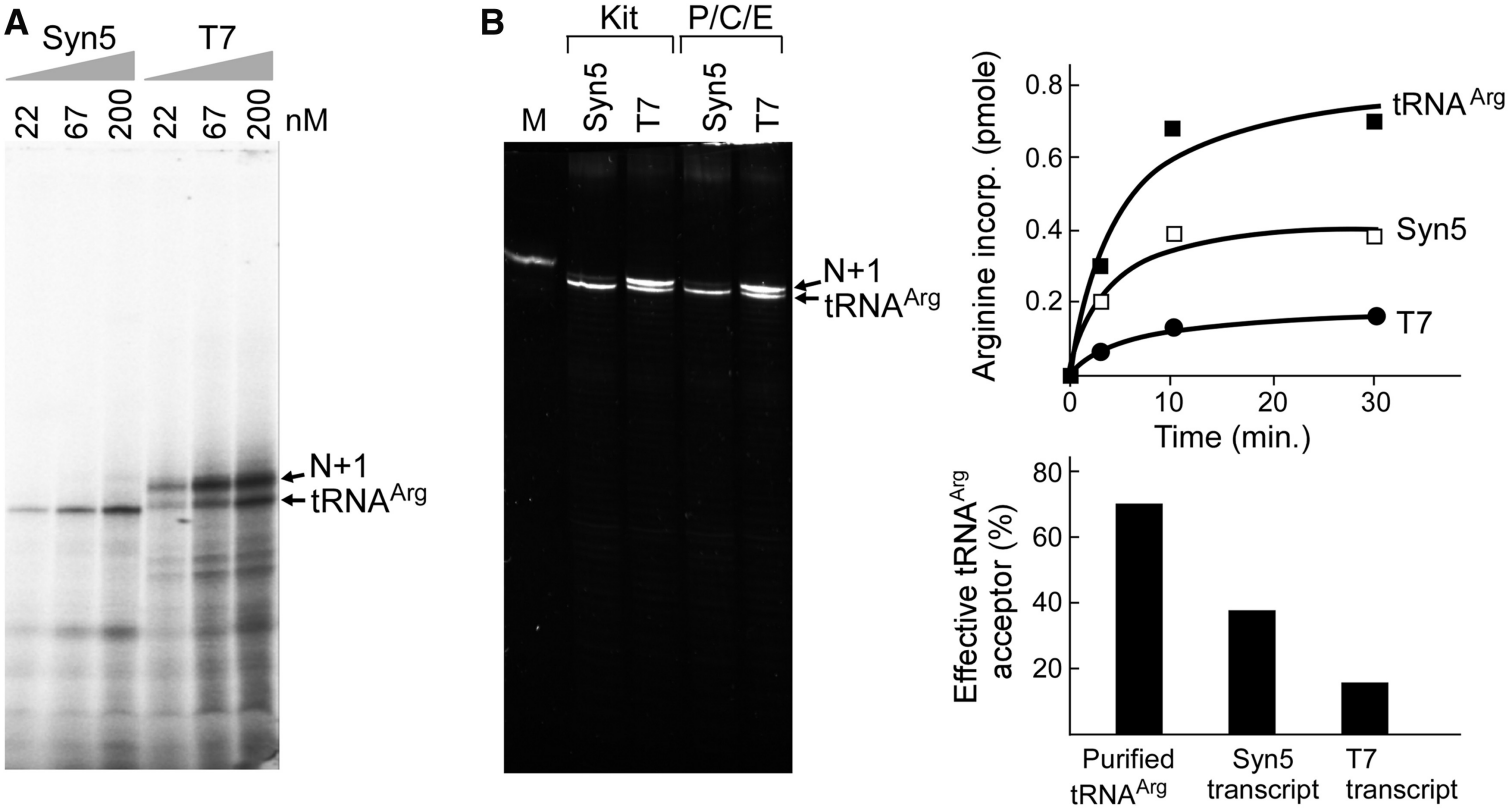
Synthesis of tRNA^Arg^ by Syn5 and T7 RNA polymerase. (**A**) Synthesis of tRNA^Arg^ on an analytical scale using varying amounts of Syn5 and T7 RNA polymerase. Reactions were carried out in the presence of radioactively labeled CTP, and either 22, 67 or 200 nM of either Syn5 or T7 RNA polymerase at 37°C. Bands corresponding to the tRNA^Arg^ and N + 1 products are indicated on the right. (**B**) Synthesis of tRNA^Arg^ on a preparative scale using Syn5 and T7 RNA polymerase. The denaturing gel shows an analysis of an aliquot of total RNA produced by Syn5 and T7 RNAP at 37°C in a 200 µl reaction. RNAs were purified by either ZYMO RNA clean kit or phenol/chloroform extraction followed by ethanol precipitation (P/C/E), as noted on top of the gel. The marker (M) in the left lane contains native tRNA^Arg^ (containing modified bases) that had been overexpressed and purified from *E. coli* cells. The two major bands in the total RNAs are tRNA^Arg^ and the N + 1 product as marked. The inset graph shows the charging of arginine on tRNA^Arg^ from 0.2 µM total RNAs (purified native tRNA^Arg^, Syn5 RNA polymerase products or T7 RNA polymerase products) by *E. coli* total aminoacyl-tRNA synthetases as a function of time. The maximum charging capacity at 30 min was converted into the amount of functional tRNA^Arg^ molecules (arginine and tRNA^Arg^ are at a 1:1 ratio in the aminoacylation reaction). Based on this conversion, the percentage of functional tRNA^Arg^ molecules in the total RNA transcripts in each reaction was calculated and shown in the column chart at the bottom right.

To further investigate N + 1 product synthesis by Syn5 and T7 RNA polymerases (Figure 4), reaction mixtures (10 µl, Figure 4A) containing 40 mM Tris-HCl pH 8.0, 6 mM MgCl_2_, 2 mM spermidine, 10 mM DTT, 200 µM ATP, GTP and UTP, 10 µM [α-^32^P]CTP, 1.5 U/µl RNaseOUT^TM^ recombinant ribonuclease inhibitor, 0.5 µM of DNA templates S5P-tDNAR or T7P-tDNAR, and varying amounts of Syn5 or T7 RNA polymerase were incubated at 37°C for 30 min before adding of one unit of DNase I to each reaction mixture, and then incubated for an additional 15 min at 37°C. Reactions were then terminated by the addition of 4 µl of loading dye containing 95% formamide and 40 mM EDTA. Samples were then heated at 90°C for 1 min and 4 µl of each sample was loaded onto a 6% TBE-Urea denaturing polyacrylamide gel. After electrophoresis, gels were dried and analysed using a Fuji BAS 1000 Bioimaging Analyzer. For the results shown in Figure 4B, 200 µl reaction mixtures containing 40 mM Tris-HCl pH 8.0, 6 mM MgCl_2_, 2 mM spermidine, 10 mM DTT, 600 µM ATP, GTP CTP and UTP, 1.5 U/µl RNaseOUT^TM^ recombinant ribonuclease inhibitor, 0.04 U/µl yeast inorganic pyrophosphatase, 0.5 µM of DNA templates S5P-tDNAR or T7P-tDNAR, and 100 nM Syn5 or T7 RNA polymerase. Reaction mixtures were incubated at 37°C for 2 h, followed by the addition of 10 unit of DNase I to each reaction mixture and an additional incubation of 20 min at 37°C. RNA products were then purified with RNA Clean & Concentrator™-5 kit or traditional phenol-chloroform extraction/ethanol precipitation with resuspension into 30 µl H_2_O. RNA was quantified by measurement of OD_260_ using NANOPHOTOMETER (IMPLEN). Syn5 RNA polymerase reaction mixture (5 µl) and T7 RNA polymerase reaction mixture (1 µl) were mixed with denaturing dye, heated at 90°C for 1 min and loaded onto a 15% TBE-Urea denaturing polyacrylamide gel. RNAs were visualized by staining with ethidium bromide. Native tRNA_Arg_ purified from *E. coli* (20) was loaded as a marker.

**Figure 5.**
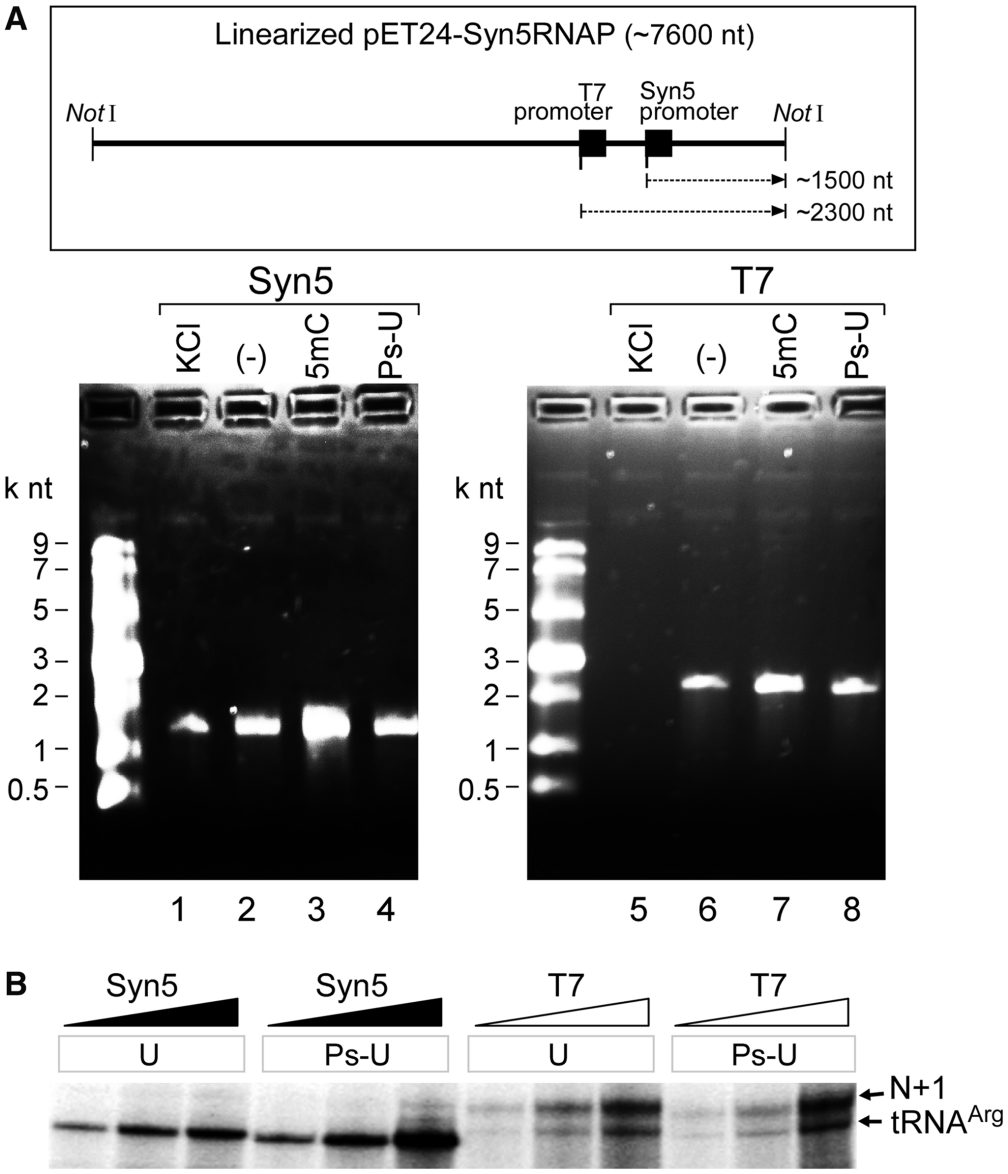
Synthesis of long run-off products and the incorporation of modified nucleosides by Syn5 and T7 RNA polymerases. (**A**) The plasmid template shown in the schematic was linearized with the restriction enzyme NotI. Transcripts produced by 10 nM of either Syn5 or T7 RNA polymerase were analysed on an agarose gel. Lane 1 shows the products of reactions in the presence of 160 mM KCl. Lane 2 shows the products of the same reactions carried out in the absence of KCl. Lanes 3 and 4 show the products of reactions where CTP was replaced by 5mCTP (lane 3) and UTP by Ps-UTP (lane 4) in the absence of KCl. (**B**) Incorporation of UTP or Ps-UTP into small RNA by 22, 67 and 200 nM Syn5 and T7 RNA polymerase as analysed by denaturing TBE PAGE. The region of the gel where full-length run-off products and N + 1 products migrate is shown.

In order to examine the incorporation of modified nucleotides by Syn5 and T7 RNA polymerases (Figure 5), reaction mixtures (10 µl, Figure 5A) contained 40 mM Tris-HCl pH 8.0, 6 mM MgCl_2_, 2 mM spermidine, 10 mM DTT, 600 µM ATP, GTP, CTP (or 5Me-CTP) and UTP (or pseudo-UTP), 1.5 U/µl RNaseOUT^TM^ recombinant ribonuclease inhibitor, 0.04 U/µl yeast inorganic pyrophosphatase, 10 nM linearized (NotI treated) pET24-Syn5RNAP, plus or minus 160 mM KCl, and 10 nM Syn5 or T7 RNA polymerase. Reactions with Syn5 RNAP were carried out at 24°C and reactions with T7 RNAP were carried out at 37°C. RNA products were then purified with RNA Clean & Concentrator™-5 kit using a final solution of 10 µl, and 5 µl of each sample was mixed with 5 µl denaturing RNA loading dye. Samples were then heated at 65°C for 5 min and loaded onto a 1.6% TAE agarose gel. RNA products were visualized by staining with ethidium bromide. For Figure 5B, reaction mixtures (10 µl) containing 40 mM Tris-HCl pH 8.0, 6 mM MgCl_2_, 2 mM spermidine, 10 mM DTT, 200 µM ATP, GTP and UTP (or pseudo-UTP), 10 µM [α-^32^P]CTP, 1.5 U/µl RNaseOUT^TM^ recombinant ribonuclease inhibitor, 0.5 µM of DNA templates S5P-tDNAR or T7P-tDNAR, and varying amounts of Syn5 or T7 RNAP. Reaction mixtures were incubated at 24°C (for Syn5 RNAP) or 37°C (for T7 RNA polymerase) for 30 min before adding one unit of DNase I to each reaction mixture and incubation for an additional 15 min at 37°C. Reactions were then terminated by the addition of 4 µl of loading dye containing 95% formamide and 40 mM EDTA. Samples were then heated at 90°C for 1 min and 4 µl of each sample was loaded onto a 6% TBE-Urea denaturing polyacrylamide gel. After electrophoresis, gels were dried and analysed using a Fuji BAS 1000 Bioimaging Analyzer.

**Figure 6.**
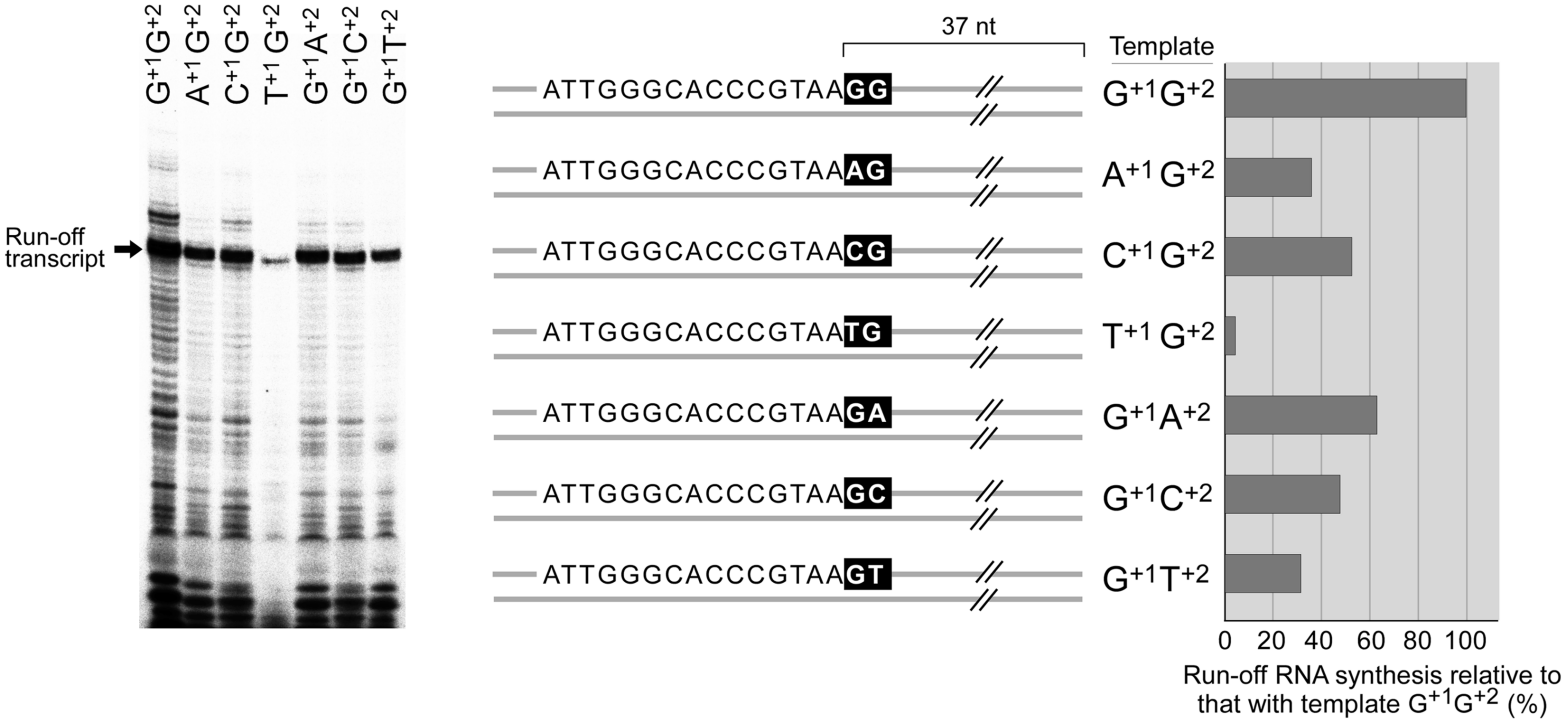
Preference of initiating nucleotides by Syn5 RNA polymerase. Various DNA templates (as shown schematically) directing the synthesis of transcripts differ by only one initiating nucleotide at the first or second position were transcribed by Syn5 RNA polymerase. The transcripts were separated on a 10% TBE-urea gel and the amount of a 37 nt run-off RNA product (arrow) was quantified and compared as shown in the chart.

In order to determine if Syn5 RNA polymerase prefers specific nucleotides to initiate RNA synthesis at the promoter we used DNA templates containing the promoter but with different nucleotides at the initiation site (Figure 6). The reactions (10 µl) contained 40 mM Tris-HCl pH 8.0, 6 mM MgCl_2_, 2 mM spermidine, 10 mM DTT, 200 µM GTP, CTP and UTP, 10 µM [α-^32^P]ATP, 1.5 U/µl RNaseOUT^TM^ recombinant ribonuclease inhibitor, 1 µM DNA templates (as specified in Figure 6), 100 nM Syn5 or T7 RNA polymerase. Incubation was carried out at 24°C for 30 min before the addition of one unit of DNase I to each reaction mixture, and then incubation was continued for 15 min at 37°C. Reactions were terminated by the addition of 4 µl of denaturing loading dye and then heating at 90°C for 1 min. Each sample (4 µl) was loaded onto a 10% TBE-Urea denaturing polyacrylamide gel. After electrophoresis, gels were dried and the radioactivity was analysed using a Fuji BAS 1000 Bioimaging Analyzer.

**Figure 7.**
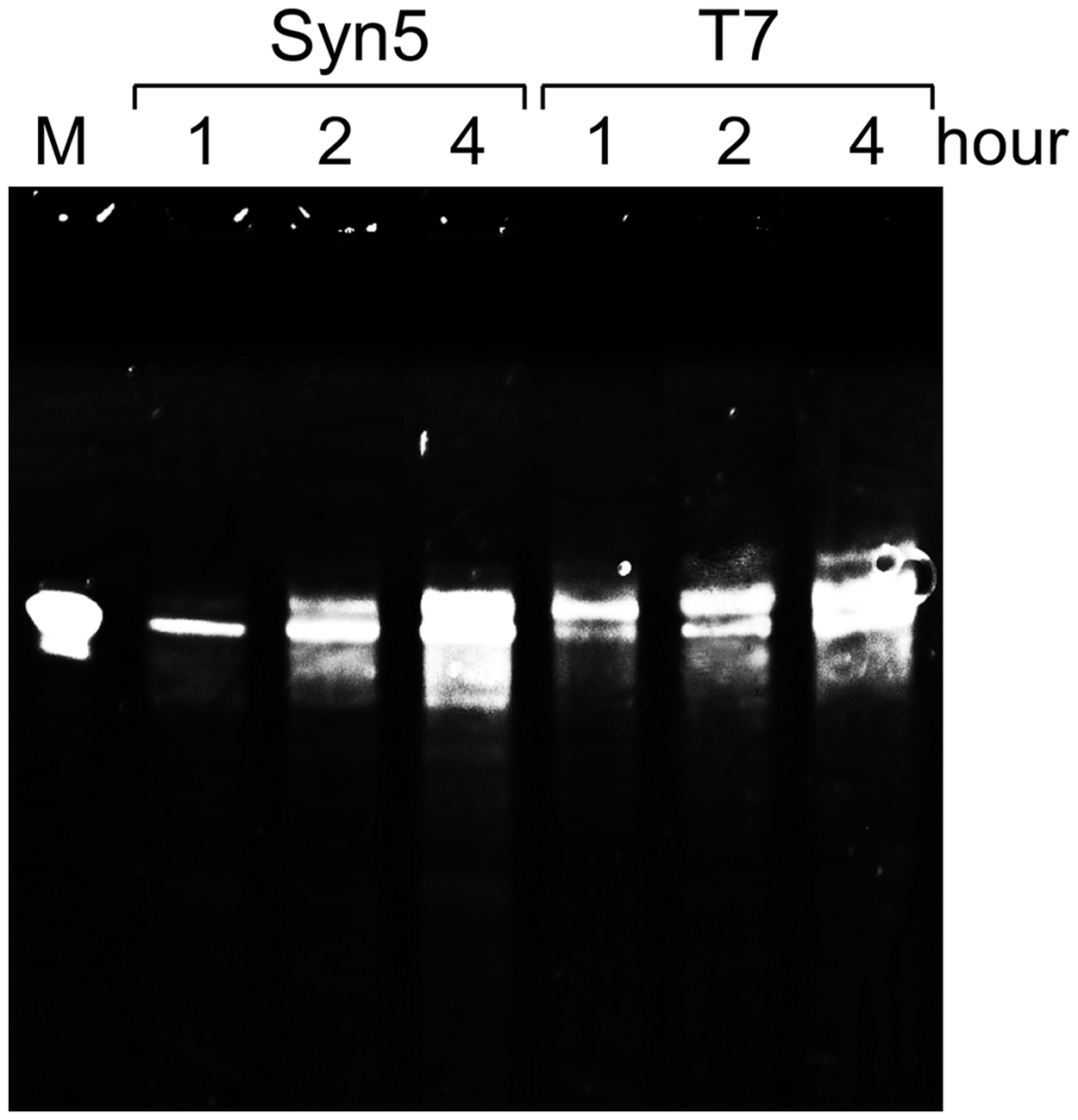
Stability of Syn5 RNA polymerase during prolonged incubations. Syn5 or T7 RNA polymerase reaction was carried out at 37°C. After 1, 2 and 4 h their products were separated on a 15% TBE-urea gel and the run-off tRNA transcripts were shown as white bands by ethidium bromide staining.

To examine the stability of Syn5 RNA polymerase over long incubation times (Figure 7), reaction mixtures (10 µl) contained 40 mM Tris-HCl pH 8.0, 6 mM MgCl_2_, 2 mM spermidine, 10 mM DTT, 600 µM ATP, GTP, CTP and UTP, 1.5 U/µl RNaseOUT^TM^ recombinant ribonuclease inhibitor, 0.04 U/µl yeast inorganic pyrophosphatase, 2 µM DNA templates S5P-tDNAR or T7P-tDNAR, and 200 nM Syn5 or T7 RNA polymerase. After incubations for 1, 2 and 4 h at 37°C, one unit of DNase I was added to each reaction mixture and incubation was continued for 15 min at 37°C to remove the DNA templates. Denaturing loading dye (4 µl) was then added to the mixture. Samples were heated at 90°C for 1 min and were loaded onto a 15% TBE-Urea denaturing polyacrylamide gel. RNA products were visualized by staining with ethidium bromide.

### tRNA aminoacylation assay

tRNA aminoacylation assay was performed as described previously (21). Reaction mixtures containing 100 mM Tris-HCl, pH 7.5, 30 mM KCl, 12 mM MgCl_2_, 0.5 mM DTT, 4 mM ATP, 0.2 µM RNA (purified native *E. coli* tRNA^Arg^ (20) or purified tRNA^Arg^ transcripts from Syn5 or T7 RNA polymerase reaction using RNA Clean & Concentrator™-5 kit), 15 µM [^3^H] arginine (15 Ci/mmol) and 4 µM *E. coli* total aminoacyl-tRNA synthetases were incubated at 37°C for the indicated times. Each reaction mixture (5 µl) was spotted onto Whatman DE(81) filters and the reactions were terminated by washing the filters in 10% TCA solution containing 0.5 mM arginine. The filters were washed twice with 10% TCA and once with ethanol, and then dried. The amount of amino acid charged to tRNA was determined by measuring the radioactivity remaining bound to the filters.

## RESULTS AND DISCUSSION

### Purification of Syn5 RNA polymerase

Our previously reported three-step purification of Syn5 RNA polymerase (18) from *E. coli* cells yielded active enzyme suitable for preliminary biochemical characterization and identification of its promoter. However, the low solubility and yield of protein, the tedious purification procedure and the contamination of nucleases led us to improve the purification procedure. Based on the properties of other marine viral proteins we have been characterizing we have modified and improved the purification procedure for Syn5 RNA polymerase. We improved the yield of protein more than 10-fold by using an unusually high concentration of NaCl (2 M) during chromatography (for details see ‘Materials and Methods’ section). Additional benefits of this procedure are that the time required for purification is reduced by a factor of two and the specific activity of the enzyme is increased 2-fold (Figure 1). The presence of 2 M NaCl significantly improves the separation between Syn5 RNA polymerase and *E. coli* nucleases, as the Ni-NTA chromatography step alone yields apparently homogenous enzyme (Figure 1A) free of nuclease activities (data not shown). The activity of the Syn5 RNA polymerase by Ni-NTA and gel filtration chromatography is close to that of T7 RNA polymerase (Figure 1B). Hereafter Syn5 RNA polymerase purified by this procedure is used in all experiments.

### Termination and apparent processivity of Syn5 RNA polymerase

The fact that the Syn5 genome has only two promoters for the Syn5 RNA polymerase suggests that the enzyme must have a high processivity (18). We compared the apparent processivity of Syn5 RNA polymerase to that of T7 RNA polymerase by constructing two plasmid templates with a single promoter for either Syn5 or T7 RNA polymerase. On such templates the RNA polymerases can perform rolling-circle synthesis to reach their maximum processivity. We found that, like most RNA polymerases but not DNA polymerases, Syn5 RNA polymerase is unable to extend an RNA primer annealed to single-stranded DNA, even if mixed with a double-stranded DNA template that contains a Syn5 promoter (data not shown), suggesting that it can only initiate synthesis at a promoter and would not reinitiate synthesis at the abortive transcript if dissociate during synthesis. Therefore, the length of transcripts synthesized by Syn5 and T7 RNA polymerase is likely a measurement of processivity. A gel assay was used to measure the processivity (Figure 2). Syn5 RNA polymerase has a specific activity similar to that of T7 RNA polymerase (Figure 2A). The product of the Syn5 RNA polymerase reaction was confirmed to be RNA by its resistance to DNase I and sensitivity to RNase I (Figure 2B). Addition of KCl increases the yield of transcripts of Syn5 RNA polymerase (Figure 2C), with the highest yield observed at 160 mM KCl, consistent with our previous report (18). However, this stimulation is only observed when circular plasmids are used as templates (described below).

The RNA polymerase products were separated under denaturing conditions. As shown in Figure 2D, the longest products synthesized by both enzymes are longer than 9 kb, with a greater amount of these larger transcripts produced by the Syn5 RNA polymerase. However, abortive products of specific patterns were observed using both polymerases. For Syn5 RNA polymerase, the abortive products are of the size of multiple rounds of the plasmid, suggesting that the clash of an elongating and initiating RNA polymerase at the promoter region of the same plasmid is the cause of synthesis abortion. For T7 RNA polymerase two sets of abortive products were present, of which one is the same as that mentioned above for Syn5 RNA polymerase. Another set of abortive products starts with a band of ∼1000 nt, and then repeats at multiple integrals of the plasmid length, suggesting that a certain sequence in the plasmid terminates the T7 reaction, even though a T7-terminator-like sequence could not be found in the plasmid. Both plasmids were then linearized to allow run-off transcription (Figure 2E). As expected, both RNA polymerases synthesized a full-length run-off product of the plasmid size. T7 RNA polymerase has an additional abortive product of approximately equal intensity. We also examined the ability of a well-characterized stem-loop structure that terminates T7 RNA polymerase synthesis to terminate Syn5 RNA synthesis. No obvious termination of Syn5 RNA polymerase transcription was observed on plasmid pET28a DNA in which this structure is imbedded (Figure 2F). While as expected, ∼70% of T7 transcription is terminated by this signal (Figure 2F). To confirm the identity of the abortive products synthesized by Syn5 RNA polymerase, varying amounts of RNA polymerase were incubated with the same amount of template. If our model is correct that it is the clash of multiple enzymes at the promoter that is the basis for transcription abortion, then decreasing the amount of enzyme should result in fewer abortive products. Indeed, lowering the concentration of either Syn5 or T7 RNA polymerase reduces one set of abortive products (Figure 2G). However, another set of abortive products remains with T7 RNA polymerase, even at low concentrations of enzyme (Figure 2H), confirming that these products are the result of sequence-dependent terminations. Such undesired termination products presents one of the major drawbacks of using T7 RNA polymerase for the synthesis of run-off transcripts.

For reactions that contained low concentrations of Syn5 RNA polymerase, most of the products were over 9 kilo nt with some approaching 30 kilo nt (Figure 2F). The greater apparent processivity of Syn5 RNA polymerase compared with T7 RNA polymerase is consistent with the promoter distribution in the two phage genomes: Syn5 RNA polymerase uses a single promoter to transcribe over 37 kb, while T7 requires 15 promoters to transcribe its genome that is of similar size to that of Syn5 (Figure 2G).

### Syn5 versus T7 RNA polymerase in run-off transcription

Parameters influencing run-off transcription reactions catalysed by Syn5 and T7 RNAP were investigated using defined dsDNA templates. The DNA oligonucleotides contain the sequence encoding *E. coli* 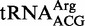 directly downstream of a Syn5 or T7 RNA polymerase promoter that allowed us to analyse the function of the run-off products. We could not detect a significant amount of N + 1 product band in reactions containing Syn5 RNA polymerase products under all conditions tested (Figure 3A). On the other hand, the products produced using T7 RNA polymerase contained a large amount of the N + 1 product. Syn5 RNA polymerase also demonstrated much better activity at lower temperatures and elevated salt concentrations. Only at 37°C in the absence of salt does T7 RNA polymerase produce a higher yield of run-off product than Syn5 RNA polymerase. T7 RNA polymerase is known to be inhibited by high salt; little product is produced with T7 RNA polymerase at 160 mM KCl while even at 250 mM KCl Syn5 RNA polymerase produces significant amounts of product. We also prepared His-tagged T7 RNA polymerase and compared it to the His-tagged Syn5 RNA polymerase (Figure 3B). The presence of the His tag on T7 RNA polymerase had no effect on its properties with regard to synthesis of the N + 1 product.

### Synthesis of active tRNA molecules by Syn5 and T7 RNA polymerase

In order to determine the effect of RNA polymerase concentration on the homogeneity of transcription products, varying amounts of Syn5 and T7 RNA polymerases were used to synthesize tRNA. A reaction temperature of 37°C was used since T7 RNA polymerase synthesizes more homogeneous products at this temperature. Under identical reaction conditions except for the promoter sequences, Syn5 RNA polymerase consistently produced very low amounts of N + 1 product (Figure 4A). In contrast, at all concentrations of T7 RNA polymerase, more N + 1 product was produced than the desired product.

We also compared the ability of both enzymes to synthesize tRNA on a preparative scale (Figure 4B). Under the reaction conditions described in ‘Materials and Methods’ section, RNAs were recovered from 200 µl Syn5 and T7 RNA polymerase reactions using either a RNA Clean & Concentrator™-5 kit or by phenol-chloroform extraction/ethanol precipitation. Analysis of both products on a sequencing gel again confirmed that T7 RNA polymerase produced N + 1 products in 3-fold excess over the desired product, while Syn5 RNA polymerase produced N + 1 products <10% of the desired product. To test the quality of transcripts by either enzyme, tRNA function of the purified RNAs was measured in an aminoacylation assay. Only tRNAs with a precise 3′-CCA terminus can accept the cognate amino acid. Using an excess of *E. coli* aminoacyl-tRNA synthetases and radioactive labeled arginine, we found that the amino acid charging activity of the tRNA products produced using Syn5 RNA polymerase products is much higher than those obtained using T7 RNA polymerase. This result confirms that the predominant product from the Syn5 RNA polymerase reaction is tRNA of precise length while that from the T7 RNA polymerase reaction is undesired RNA, most likely N + 1 product. About 40% of the tRNA produced by Syn5 RNA polymerase is active, compared with 15% for that produced by T7 RNA polymerase. In the current study we did not carry out gel purification but instead used an RNA purification kit/or phenol-chloroform extraction. Consequently, the purified RNA products contain other species of shorter RNAs probably resulting from degradation and longer extended products. We believe that a gel extraction can efficiently remove most of the other species. However, it would not be possible to separate the N + 1 product from tRNA by gel extraction due to the limited separation of products with 1 nt difference on PAGE gel, especially when large amounts of RNA are loaded on the gel.

### Incorporation of modified nucleotides

Some applications use RNA that contains modified nucleosides to mimic functional RNA molecules or to improve the function of RNA. For instance, 5-methylcytidine (5 mC) and pseudouridine (Ps-U) are introduced into mRNA to improve its function *in vivo* (22). We examined the efficiency by which Syn5 RNA polymerase incorporated 5mC and Ps-U into a 1500 nt run-off RNA transcript (Figure 5). When CTP or UTP was replaced by 5mCTP or Ps-UTP in the reaction, the yield of RNA, based on A_260_, was 0.76 and 0.63 µg, respectively, comparable to 0.69 µg when CTP and UTP were present (Figure 5A). T7 RNA polymerase also incorporates 5mC and Ps-U at comparable efficiencies, with the yield of RNA being 0.64, 0.75 or 0.59 µg when CTP/UTP, 5mCTP or Ps-UTP was present, respectively. The yield of RNA by each RNA polymerase at their optimal temperatures was comparable (0.69 µg versus 0.64 µg). T7 RNA polymerase was completely inhibited by 160 mM KCl while Syn5 RNA polymerase still produced 0.31 µg RNA, ∼50% of that produced in the absence of salt. The efficiency of incorporation of Ps-U into small RNA transcripts by both RNAPs was also investigated (Figure 5B). Using radioactively labeled CTP, the yield of tRNA^Arg^ was higher for both RNAPs when UTP was replaced by Ps-UTP, with T7 RNA polymerase again producing predominantly the N + 1 product.

### Initiating nucleotides for transcription by Syn5 RNA polymerase

T7 RNA polymerase has a stringent requirement for GTP as the initiating nucleotide and a strong preference for GG as the initiating sequence (4,23). We find that Syn5 RNA polymerase also prefers a GG initiating sequence on the transcript for a maximum yield of run-off transcripts (Figure 6). However, its preference for the initiating nucleotide is less stringent than that of T7 RNA polymerase. When the template sequences are modified to change the encoding first nucleotide (+1 position) of the transcript from G to A or C, the yield of run-off product drops to 36 and 52%, respectively. When such a change (from G to A or C or U) is at the second position of transcript (+2), the yield of run-off product drops to 64, 48 and 31%, respectively. The only non-favored initiating nucleotide (+1) is U, giving only 5% of RNA synthesis compared with a transcript starting with GG dinucleotide (Figure 6).

### Stability of Syn5 RNA polymerase

Long incubation times are often required when T7 RNA polymerase is used to obtain larger amounts of transcripts. Over a 4-h incubation at 37°C, Syn5 RNA polymerase exhibits greater stability than T7 RNA polymerase during the synthesis of run-off transcripts as judged by the amount of transcripts at 4 h (Figure 7).
